# Sphingomyelins of Local Fat Depots and Blood Serum as Promising Biomarkers of Cardiovascular Diseases

**DOI:** 10.17691/stm2024.16.1.06

**Published:** 2024-02-28

**Authors:** E.V. Belik, Yu.A. Dyleva, E.G. Uchasova, S.V. Ivanov, A.N. Stasev, M.G. Zinets, O.V. Gruzdeva

**Affiliations:** Researcher, Laboratory of Homeostasis Research, Department of Experimental Medicine; Research Institute for Complex Issues of Cardiovascular Diseases, 6 Academician L.S. Barbarash Blvd, Kemerovo, 650002, Russia; Senior Researcher, Laboratory for Homeostasis Research, Department of Experimental Medicine; Research Institute for Complex Issues of Cardiovascular Diseases, 6 Academician L.S. Barbarash Blvd, Kemerovo, 650002, Russia; Senior Researcher, Laboratory for Homeostasis Research, Department of Experimental Medicine; Research Institute for Complex Issues of Cardiovascular Diseases, 6 Academician L.S. Barbarash Blvd, Kemerovo, 650002, Russia; Leading Researcher, Laboratory of X-ray Endovascular and Reconstructive Surgery of the Heart and Vessels, Department of Cardiovascular Surgery; Research Institute for Complex Issues of Cardiovascular Diseases, 6 Academician L.S. Barbarash Blvd, Kemerovo, 650002, Russia; Senior Researcher, Laboratory of Heart Diseases, Department of Cardiovascular Surgery; Research Institute for Complex Issues of Cardiovascular Diseases, 6 Academician L.S. Barbarash Blvd, Kemerovo, 650002, Russia; Cardiac Surgeon, Department of Cardiac Surgery No.1; Research Institute for Complex Issues of Cardiovascular Diseases, 6 Academician L.S. Barbarash Blvd, Kemerovo, 650002, Russia; Associate Professor, Professor of the Russian Academy of Sciences, Head of the Laboratory for Homeostasis Research, Department of Experimental Medicine; Research Institute for Complex Issues of Cardiovascular Diseases, 6 Academician L.S. Barbarash Blvd, Kemerovo, 650002, Russia; Head of the Department of Medical Biochemistry; Kemerovo State Medical University, 22A Voroshilov St., Kemerovo, 650056, Russia

**Keywords:** sphingomyelin, epicardial adipose tissue, perivascular adipose tissue, coronary artery disease, acquired degenerative valvular heart disease

## Abstract

Assessment of the blood lipid spectrum does not always properly reflect local dysfunctional changes in the adipose tissue and prevents identification of all patients at high risk of cardiovascular diseases (CVD). Monitoring of changes in sphingomyelin levels allows to assess and anticipate the development and/or severity of these diseases, as well as to make sphingomyelins new therapeutic targets.

**The aim of the study** was to evaluate the sphingomyelin spectrum of local fat depots and blood serum in connection with clinical and instrumental indicators in patients with coronary artery disease (CAD) and patients with degenerative acquired valvular heart disease (AVHD).

**Materials and Methods:**

The study analyzed samples of subcutaneous, epicardial, perivascular adipose tissue (SAT, EAT, PVAT, respectively) received from 30 patients with CAD and 30 patients with AVHD. Sphingomyelin spectrum of the blood serum was assessed using a high-resolution chromatography-mass spectrometric complex (liquid chromatograph of the Agilent 1200 series (Agilent Technologies, USA) with a maXis impact mass spectrometric detector (Bruker Daltonics, Germany)). Determination of the levels of sphingomyelins (SM) in adipose tissue samples was conducted by high performance liquid chromatography with mass spectrometric detection in the mass/charge ratio range from 100 to 1700.

**Results:**

Consistent sphingomyelin spectrum of local fat depots and blood serum was revealed in CAD and AVHD. However, the content of SM varied: in CAD, a specific enhancement of SM in epicardial adipose tissue was observed compared to subcutaneous and perivascular localization. In AVHD, PVAT was characterized by a statistically significant increase in the levels of all SM relative to EAT. Almost all measured SM types in the serum of patients with CAD were higher than the levels in the AVHD group.

**Conclusion:**

Established associations of indicators of the sphingomyelin profile of adipose tissue and blood serum with clinical and instrumental indicators in CVD indicate the relationship between the metabolism of SM in adipose tissue of cardiac localization and disorders of systolic and diastolic function of the LV in patients with CVD, multivessel coronary disease in CAD and allow the use of SM as promising biomarkers of CVD. However, further research is needed to clarify the nature of these relationships.

## Introduction

It is well known that lipid metabolism disorders play a central role in development of cardiovascular diseases (CVD). Although determination of total cholesterol/ cholesterol (TC), high-, low-, and very low-density lipoprotein cholesterol/cholesterol (HDL-C, LDL-C, VLDL-C, respectively) and triacylglycerides ensures an acceptable estimate of adverse cardiovascular events probability, the limited evidence proves a high residual risk upon the achievement of the target values of these indicators, because other lipids can also act as cholesterol-independent disease factors [1]. Currently, scientists’ attention is paid mainly to the study of the lipid spectrum of plasma/serum, but this assessment does not always properly reflect local dysfunctional changes in the adipose tissue (AT) thus preventing identification of all patients at high risk of CVD [1].

Progress in lipidomics over the past 20 years has contributed to active study of AT metabolic dysregulation in CVD. However, there is practically no information about the lipid content of epicardial (EAT) and perivascular adipose tissue (PVAT), which are closest to the affected area, although local cardiac fat depots are metabolically active and can lead to development/ progression of the coronary artery disease (CAD) due to paracrine release of proatherogenic mediators [2, 3].

The most interesting biologically active lipids are sphingolipids, which regulate numerous cellular functions and are associated with CVD. Sphingomyelins (SM) are specific substances consisting of a phosphocholine head group, sphingosine, and a fatty acid [4].

Sphingomyelins are formed from ceramides synthesized in the endoplasmic reticulum through sphingomyelin synthase. The SM enzyme belongs to the phospholipase group and can be expressed with several isoforms: SM1 (encoded by the *SGMS1* gene) is responsible for the SM synthesis of in the Golgi apparatus, SM2 (*SGMS2*) is localized mainly in the plasma membrane [4].

Sphingomyelin is considered a most common sphingolipid in human AT and serum [5]. For instance, SM accounts for about 87% of the total serum sphingolipids [6]. Earlier studies demonstrated that increased levels of serum SM are a risk factor for CVD [7]. Moreover, correlations between an increase in SM and insulin sensitivity, as well as between the CAD development and obesity, were identified [8]. However, the sources of excess serum SM levels have not yet been defined.

Despite numerous attempts to identify SM and determine their critical level, biological functions, means of transportation, synthesis control, and detection of SM-dependent signaling pathways in CVD, currently the data on the SM spectrum in human AT is practically unavailable [9], hence monitoring of changes in SM levels will allow to assess and anticipate the severity and/or development of CVD, as well as to make them new potential targets for therapeutic treatment. Therefore, control of the sphingomyelin spectrum may be a promising strategy for treatment of cardiovascular and metabolic diseases.

**The aim of the study** was to evaluate the sphingomyelin spectrum of local fat depots and blood serum in connection with clinical and instrumental indicators in patients with coronary artery disease and patients with degenerative acquired valvular heart disease.

## Materials and Methods

### Characteristics of patients

The sphingomyelin spectrum of local fat depots and blood serum was determined in 30 patients with CAD and 30 patients with degenerative acquired valvular heart disease (AVHD) and namely with stenosis/insufficiency of the aortic and mitral valves. The study protocol was approved by the local ethics committee of the Research Institute for Complex Issues of Cardiovascular Diseases (Kemerovo, Russia). Patients were selected in accordance with the inclusion and exclusion criteria, following GOST R 52379—2005 (Good Clinical Practice) and the principles of the Declaration of Helsinki of the World Medical Association (Ethical Principles for Medical Research Involving Human Subjects), as amended in 2013, as well as the Guidelines on Clinical Practice in the Russian Federation, approved by Order of the Ministry of Healthcare of the Russian Federation No.266 dated June 19, 2003.

All patients had indications for open cardiac surgery — direct myocardial revascularization by coronary artery bypass grafting or cardiac valve surgery. The study did not include patients over 75 years with clinically significant concomitant pathologies (diabetes mellitus of types 1 and 2, myocardial infarction, anemia, renal and liver failure, oncological and infectious inflammatory diseases in acute stage, and autoimmune diseases). The inclusion criteria for patients in the comparison group were verified AVHD and consent to participation in the study.

Patients in the study groups were comparable by gender and age (see the Table). Persons with coronary artery disease often had such CVD risk factors as arterial hypertension, dyslipidemia, and smoking in their history. Levels of total cholesterol, LDL-C, and atherogenic index in patients with CAD exceeded the same in persons with AVHD. On the contrary, the HDL-C content was higher in the AVHD group.

**Table:** Clinical characteristics of patients with cardiovascular diseases

Indicators	Patients with CAD (n=30)	Patients with AVHD (n=30)	p
Male, n (%)	18 (60.0)	17 (56.7)	0.056
Age (years), Me [Q1; Q3]	64.9 [47.8; 69.5]	59.3 [43.7; 62.1]	0.071
Body mass index, Me [Q1; Q3]	26.4 [22.5; 30.2]	27.3 [23.4; 31.2]	0.062
Arterial hypertension, n (%)	17 (56.7)	7 (23.3)	0.002
Dyslipidemia, n (%)	13 (43.3)	3 (10.0)	0.001
Smoking, n (%)	15 (50.0)	5 (16.7)	0.0001
History of CAD, n (%)	18 (60.0)	11 (36.7)	0.038
History of myocardial infarction, n (%)	21 (70.0)	0	
Acute cerebrovascular accidents, n (%)	3 (10.0)	0	
Atherosclerosis of other systems, n (%)	5 (16.7)	0	
Angina pectoris, n (%):			
No	1 (3.3)	30(100)	
I FC	0	0	
II FC	14 (46.7)	0	0.0001
III FC	15 (50.0)	0	
IV FC	0	0	
CHF, n (%):			
I FC	12 (40.0)	7 (23.3)	
II FC	4 (13.3)	23 (76.7)	0.055
III FC	0	0	0.002
IV FC	0	0	
Atherosclerosis, n (%):			
of one CA	4 (13.3)	0	
of two CAs	1 (3.3)	0	
of three or more CAs	24 (80.0)	0	
Ejection fraction (%), Me [Q1; Q3]	53.6 [46.3; 58.9]	51.6 [42.5; 55.8]	0.046
***Lipid profile***					
TC (mmol/L), Me [Q1; Q3]	5.89 [4.12; 8.25]	3.97 [3.56; 5.75]	0.030
HDL-C (mmol/L), Me [Q1; Q3]	0.91 [0.51; 0.97]	1.20 [0.97; 1.80]	0.035
LDL-C (mmol/L), Me [Q1; Q3]	3.31 [2.95; 5.65]	2.21 [1.93; 3.20]	0.021
VLDL-C (mmol/L), Me [Q1; Q3]	0.60 [0.45; 1.33]	0.61 [0.31; 1.28]	0.137
Triglycerides (mmol/L), Me [Q1; Q3]	1.75 [1.52; 2.83]	1.32 [0.88; 2.25]	0.098
Atherogenic index, Me [Q1; Q3]	3.38 [2.75; 4.33]	2.38 [1.95; 2.72]	0.014
***Inpatient treatment***			
Aspirin, n (%)	28 (93.3)	0	
Clopidogrel, n (%)	4 (13.3)	0	
Warfarin, n (%)	0	25 (83.3)	
β-blockers, n (%)	27 (90.0)	26 (86.7)	0.091
ACEI, n (%)	23 (76.7)	24 (80.0)	0.247
Statins, n (%)	30(100)	22 (73.3)	0.059
Ca-channel blockers, n (%)	23 (76.7)	21 (70.0)	0.166
Nitrates, n (%)	1 (3.3)	2 (6.7)	0.107
Diuretics, n (%)	24 (80.0)	25 (83.3)	0.087

Note: CA — coronary artery, ACEI — angiotensin-converting enzyme inhibitors. Other keys are explained in the text.

Patients with AVHD more often suffered from chronic heart failure (CHF) of functional class (FC) II. Patients’ left ventricular (LV) systolic function was assessed using the Teicholtz LV ejection fraction; it was 53.6 [46.3; 58.9]% in patients with CAD and 51.6 [42.5; 55.8]% — in the AVHD group, which corresponded to the preserved ejection fraction.

During the in-hospital period, all patients were treated in line with the standard drug therapy in accordance with the recommendations of the Ministry of Healthcare of the Russian Federation (2020) and the European Society of Cardiology (2020) (see the Table).

During surgery (coronary bypass surgery or correction of heart defects), samples (3–5 g) of AT of subcutaneous (SAT), epicardial (EAT), and perivascular (PVAT) localization were taken, after which they were cryogenically frozen with liquid nitrogen and stored at –150°C. The SAT was sourced from the subcutaneous tissue of the lower angle of the mediastinal wound, EAT — from the area of its greatest presence (right parts of the heart — right atrium and right ventricle), PVAT — from the area of the right coronary artery.

The lipidomic composition of the patients’ blood serum and AT was determined using a high-resolution chromatography-mass spectrometry complex — an Agilent 1200 Series liquid chromatograph (Agilent Technologies, USA) with a maXis impact mass spectrometric detector (Bruker Daltonics, Germany). To determine the SM content in the blood serum and AT samples, liquid-liquid extraction was preliminary performed using chloroform, methanol, and water. SM analysis in samples was performed with the highperformance liquid chromatography (HPLC) with mass spectrometric detection within mass-to-charge ratio ranging from 100 to 1700. To identify SM, tandem mass spectrometry (MS) was performed in the dependent scanning mode with a window width of 5 Da. The source files resulting from HPLC-MS were converted using the msConvert software from the ProteoWizard 3.0.9987 software package into the open MzXml format, which contains information about the mass spectrum at any moment, and into the ms2 format, which contains information about the tandem mass spectra at a specific moment. We also used the MzMine software to isolate peaks, normalize to the total ion current, and create a table with information about the mass of the ion, its chromatographic peak area, and migration time. SM identification was performed with the LipidMatch scripts by matching the mass-to-charge ratio (m/z) values of experimental fragments with the m/z values of an *in silico* fragmentation library that contains over 500,000 lipid species covering over 60 lipid types, thus making LipidMatch one of the most comprehensive and accurate databases and nomenclature about lipid (LipidMaps). The study was conducted using the equipment of the Clinical Mass Spectrometry Center of the National Medical Research Center for Obstetrics, Gynecology and Perinatology named after Academician V.I. Kulakov of the Ministry of Healthcare of the Russian Federation (Moscow, Russia).

### Conditions of chromatographic analysis of lipid profiles in the positive and negative ion mode

The experiments were conducted using an HPLCMS/ MS system that included a maXis impact hybrid quadrupole time-of-flight mass spectrometric detector (Bruker Daltonics, Germany) and a Dionex UltiMate 3000 liquid chromatograph (Thermo Scientific, USA). The chromatographic determination of the SM lipidomic spectrum was performed with a ZORBAX SB-C18 column, 0.5×150 mm; 3.5 μm (Agilent Technologies, USA) with the Security Guard pre-column (Phenomenex, USA). The experiments were conducted under the following conditions:

1. Thermostat temperature — 50°C.
2. Composition and mode of the mobile phase: eluent A — 10 mM of ammonium acetate solution in a mixture of formic acid, water, and acetonitrile (0.1:40:60%), v/v; eluent B — 10 mM of ammonium acetate solution in a mixture of formic acid, water, acetonitrile, and isopropyl alcohol (0.1:2:8:90%), v/v.
3. Eluent flow rate — 35 μl/min.
4. Volume of the injected sample — 0.5 μl (in the positive ion mode), 1.0 μl (in the negative ion mode).
5. Needle washing solution — acetonitrile.
6. Indicators of mass spectrometric detection:
  mass-to-charge ratio range: 100–1700;
  capillary voltage — 4500 V (in the positive ion mode), 3000 V (in the negative ion mode);
  nebulizer gas pressure — 0.6 Bar;
  drying gas flow — 5 l/min;
  drying gas temperature — 200°C;
  chromatogram recording time — 25 min.

### Preparation of patient serum samples

100 μl of blood serum was added with 375 μl of purified water and 1200 μl of a chloroform:methanol mixture in the ratio of 2:1, v/v. The resulting mixture was thoroughly mixed on a V-32 multi-vortex device (Biosan, Latvia) for 10 min, then centrifuged at the rotation speed of 16,000 rpm for 10 min. Then, 555 μl of the lower organic layer was removed and 440 μl of chloroform-methanol 2:1, v/v, was added. The resulting mixture was thoroughly mixed on the V-32 multi-vortex for 5 min, then centrifuged at the rotation speed of 16,000 rpm for 10 min. Another 200 μl of the lower organic layer was removed and evaporated until dry using a MULTIVAP nitrogen flow concentrator (Organomation, USA) at the room temperature for 15 min. Then, the dry residue was dissolved in 100 μl of a mixture of isopropyl alcohol-acetonitrile in the ratio of 50:50%, v/v, the resulting mixture was again mixed on the V-32 multi-vortex for 5 min, then centrifuged at the rotation speed of 16,000 rpm within 10 min. 80 μl of the supernatant was put into an insert vial, and 10 μl of each sample was pooled to prepare a quality control sample.

### Preparation of patient adipose tissue samples

700 μl of a chloroform-methanol mixture in the ratio of 2:1, v/v, was added to a sample of homogenized adipocytes of the normalized mass local fat depots. The resulting mixture was put into an ultrasonic bath for 10 min, then added 440 μl of purified water and thoroughly mixed on the V-32 multi-vortex for 5 min, further it was centrifuged at the rotation speed of 16,000 rpm for 10 min. 200 μl of the lower organic layer was removed and 500 μl of the chloroform:methanol mixture (2:1, v/v) was added. The resulting mixture was also thoroughly mixed on the V-32 multi-vortex for 5 min, then centrifuged at the rotation speed of 16,000 rpm for 10 min. Then, 400 μl of the lower organic layer was removed and evaporated until dry using the MULTIVAP nitrogen flow concentrator (Organomation, USA) at the room temperature for 15 min. After that, the dry residue was dissolved in 1000 μl of the isopropyl alcohol:acetonitrile mixture (50:50%, v/v), the resulting mixture was mixed on the V-32 multi-vortex for 5 min, then centrifuged at the rotation speed of 16,000 rpm for 10 min. 200 μl of the supernatant was put into an insert vial, and 10 μl of each sample was pooled to prepare a quality control sample. The results were normalized to the degree of samples dilution and the sample weight.

***Statistical analysis of the results*** was conducted using the GraphPad Prism 8 software (GraphPad Software, USA). Data are shown as the median, 1^st^ and 3^rd^ quartiles. SM levels in blood serum and AT were determined in relative values. Intergroup differences were identified with nonparametric tests. To compare three independent groups, the Kruskal–Wallis test was applied, followed by pairwise comparisons using the Mann–Whitney U test. Comparisons between two independent groups were performed using the Mann– Whitney U test. Categorical variables in percentages were compared using the chi-square test or Fisher’s test. The relationship between serum SM levels, SM in AT samples and lipid spectrum indicators in patients with CVD was assessed using nonparametric Spearman rank correlation (r). The interdependence of CAD and serum SM levels was assessed by logistic regression. In all statistical analysis procedures, differences were considered statistically significant at a significance level of p<0.05.

## Results

**Figure 1.**
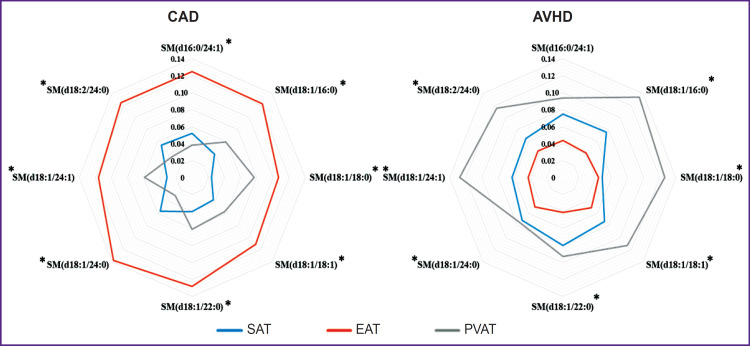
Sphingomyelin spectrum of adipose tissue of various localization in patients with cardiovascular diseases * statistically significant differences between patients with coronary artery disease and acquired heart diseases, p<0.05

During the analysis of the sphingomyelin spectrum of subcutaneous, epicardial and perivascular AT of patients with CAD and AVHD, the following types of sphingomyelins were identified: SM(d16:0/24:1), SM(d18:1/16:0), SM(d18:1/18:0), SM(d18:1/18:1), SM(d18:1/22:0), SM(d18:1/24:0), SM(d18:1/24:1), and SM(d18:2/24:0) (Figure 1). The most common sphingomyelin base was C18-sphingosine (d18:1 and d18:2). C16-sphingosine (d16:0) was also detected. At that, SM contained mainly saturated fatty acids.

Analysis of peculiarities of the SM spectrum in AT showed that EAT in patients with CAD was characterized by a higher level of all detected SM compared to subcutaneous and perivascular AT (p<0.05). In the PVAT of patients with AVHD, there was a statistically significant increase in the levels of all SM relative to EAT (p<0.05) and the levels of SM(d16:0/24:1), SM(d18:1/16:0), SM(d18:1/18:0), SM(d18:1/18:1), SM(d18:1/24:1) — relative to SAT (p<0.05).

Analysis of individual characteristics of SM in AT showed that SAT of patients with CAD was characterized by a higher level of SM with very long-chain fatty acids — SM(d16:0/24:1), SM(d18:1/24:0) — compared to PVAT (p=0.023; p=0.0026, respectively). However, the content of SM with long-chain fatty acids SM(d18:1/16:0), SM(d18:1/18:0), SM(d18:1/18:1) and with very long-chain fatty acids — SM(d18:1/22:0), SM(d18:1/24:1) — in SAT was lower than in PVAT (p=0.0001, p=0.0015, p=0.0002, p=0.006, p=0.013, respectively) (see Figure 1).

Subcutaneous AT of patients with AVHD contained more SM with long-chain fatty acids (SM(d16:0/24:1), SM(d18:1/18:1), SM(d18:1/22:0), SM(d18:1/24:0), SM(d18:1/24:1), SM(d18:2/24:0), and SM(d18:1/16:0)) than EAT. At that, EAT of patients with AVHD contained less such SM relative to PVAT (SM(d16:0/24:1), SM(d18:1/16:0), SM(d18:1/18:0), SM(d18:1/18:1), SM(d18:1/22:0), SM(d18:1/24:0), SM(d18:1/24:1), SM(d18:2/24:0)) and SAT ((SM(d16:0/24:1), SM(d18:1/16:0), SM(d18:1/18:1), SM(d18:1/22:0), SM(d18:1/24:0), SM(d18:1/24:1), SM(d18:2/24:0)).

Assessment of internosological differences in the sphingomyelin spectrum of local fat depots demonstrated a statistically significant decrease in the level of the detected SM in SAT, except for SM(d16:0/24:1) — this was seen in patients with CAD more often than in patients with AVHD. EAT of patients with CAD showed an increase in the levels of all detected SM compared to the AVHD group. On the contrary, PVAT of patients with CAD contained less SM than the same in the AVHD group.

**Figure 2.**
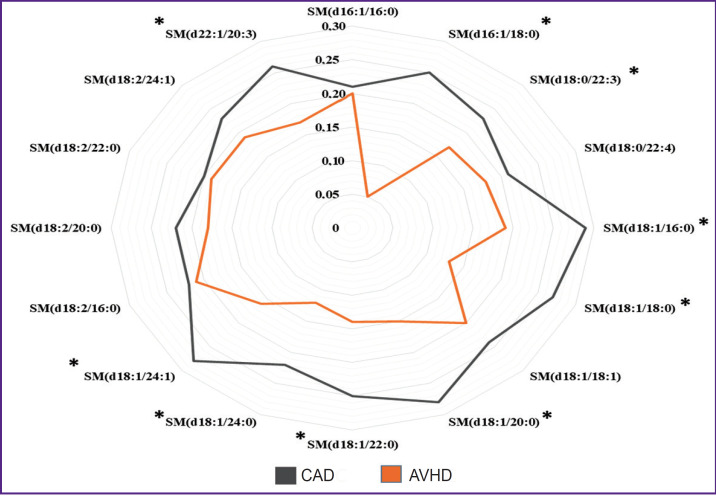
Serum SM levels in patients with cardiovascular diseases * statistically significant differences between patients with coronary artery disease and acquired heart diseases, p<0.05

The following sphingomyelin spectrum was found in the blood serum of patients with CAD and AVHD: SM(d16:1/16:0), SM(d16:1/18:0), SM(d18:0/22:3), SM(d18:0/22:4), SM(d18:1/16:0), SM(d18:1/18:0), SM(d18:1/18:1), SM(d18:1/20:0), SM(d18:1/22:0), SM(d18:1/24:0), SM(d18:1/24:1), SM(d18:2/16:0), SM(d18:2/20:0), SM(d18:2/22:0), SM(d18:2/24:1), SM(d22:1/20:3). The detected SM contained long and very long chain fatty acids. Here, the levels of almost all measured types of serum SM were statistically significantly higher in patients with CAD compared with the levels in the AVHD group (p<0.05) or trended higher (p>0.05). The most common base of serum sphingomyelins, similar to AT, was C18-sphingosine (d18:0, d18:1, and d18:2), C16-sphingosine (d16:1) was also identified. Moreover, the patients’ blood serum, in contrast to local fat depots, contained C22-sphingosine (d22:1) (Figure 2).

Most types of serum SM contained various saturated acyl chains (C16:0, C18:0, C20:0, C22:0, and C24:0), similar to local fat depots. Conspicuous is the fact that serum SM (C20:3, C22:3, C22:4) contained polyunsaturated fatty acids, which was not seen in AT.

Thus, SM was detected in the blood serum of patients with CVD, in contrast to local fat depots; this SM’s base contained C22-sphingosine (d22:1), as well as polyunsaturated fatty acids (C20:3, C22:3, C22:4). The following SM were found both in the blood serum of patients and in their local fat depots: SM(d18:1/16:0), SM(d18:1/18:0), SM(d18:1/18:1), SM (d18:1/22:0), SM(d18:1/24:0), SM(d18:1/24:1); their base was C18- sphingosine (d18:1) (see Figures 1 and 2). Based on the identified differences, one can assume that the sphingomyelin profile of blood serum corresponds (reflects) the SM content in AT only in part.

**Figure 3.**
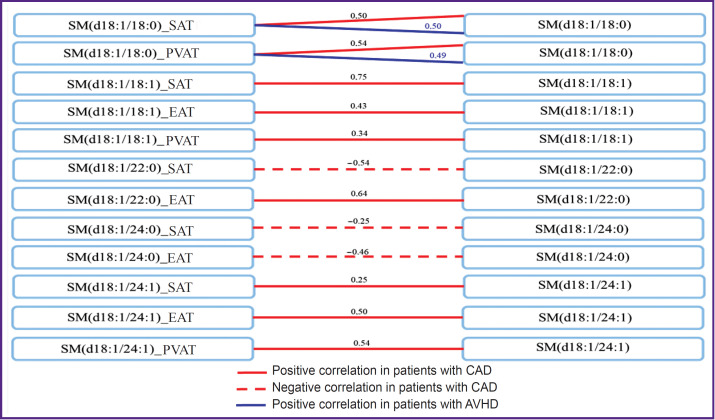
Relationship between sphingomyelins in local fat depots and their serum levels in patients with cardiovascular diseases

Levels of SM in AT and serum samples correlated with each other mainly in patients with CAD (Figure 3).

**Figure 4.**
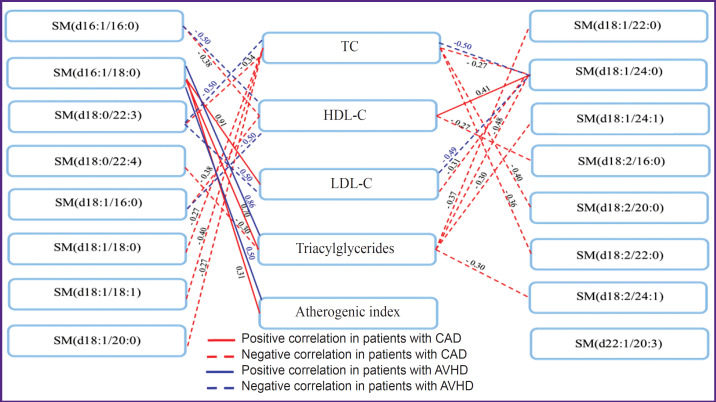
Relationship between serum sphingomyelins and lipid spectrum indicators in patients with cardiovascular diseases

The study revealed negative relationships between serum long-chain SM, having a cardioprotective effect, and TC, LDL-C, and triacylglycerides. C16-sphingosine, being the most dangerous for the cardiovascular system, negatively correlated with HDL-C and the atherogenic index in CAD. In AVHD, similar correlations were found in decreasing quantities (Figure 4).

**Figure 5.**
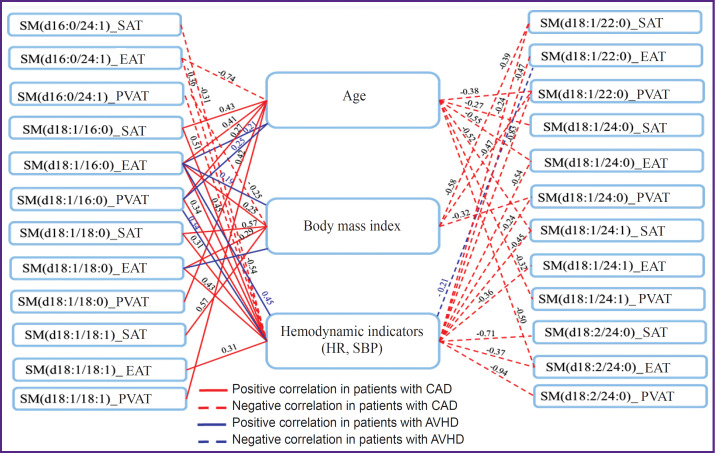
Relationship between sphingomyelins in local fat depots and clinical indicators in patients with cardiovascular diseases

In coronarogenic pathology, SM, which included a residue of C16 and C18 ceramides that have adverse cardiovascular effects, positively correlated with anthropometric (age, body mass index) and hemodynamic (heart rate, systolic blood pressure) indicators, and negatively with HDL-C, compared to persons with heart diseases, in whom only single correlations were found (Figure 5).

**Figure 6.**
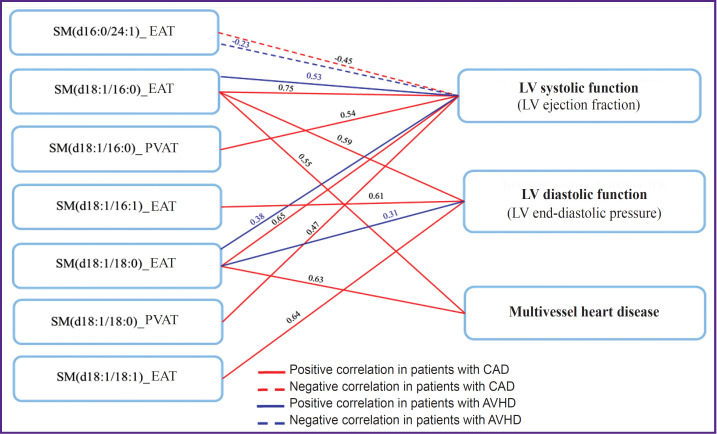
Relationship between sphingomyelins in local fat depots and instrumental indicators in patients with cardiovascular diseases

The results of the correlation analysis let one assume a relationship between the SM metabolism in AT of cardiac localization and disorders in systolic (ejection fraction) and diastolic (end-diastolic pressure — EDP) functions of the LV in patients with CVD, as well as with multivessel coronary affected areas in people with CAD (Figure 6). Here, further research is required to clarify the nature of these relationships.

The logistic regression analysis allowed to determine that CAD was associated with higher serum levels of SM(d18:1/16:0) — OR=2.63; 95% CI: 2.05–3.31; p=0.003; SM(d18:1/18:0) — OR=1.85; 95% CI: 1.52– 2.11; p=0.021; SM(d18:1/20:0) — OR=1.63; 95% CI: 1.33–2.0; p=0.001; SM(d18:1/22:0) — OR=2.0; 95% CI: 1.55–2.51; p=0.002; SM(d18:1/24:0) — OR=2.17; 95% CI: 1.75–2.71; p=0.011; SM(d18:1/24:1) — OR=2.45; 95% CI: 1.92–2.98; p=0.013.

## Discussion

The study results showed that the epicardial AT in patients with CAD and perivascular AT in people with AVHD contained the highest quantities of SM compared to other local fat depots. The most common SM base in AT was C18-sphingosine (d18:1 and d18:2), SM C16-sphingosine (d16:0) was also detected, which is consistent with the literature [4, 5].

The excess SM level in EAT in patients with CAD corresponded to an increase in serum SM compared to similar indicators in the AVHD group. At that, the majority of serum SM contained saturated acyl chains (C16:0, C18:0, C20:0, C22:0, and C24:0).

It is assumed that the saturation of the SM acyl chains is important in mediating biological/pathological characteristics of SM. For instance, Hanamatsu et al. [8] demonstrated that SM with saturated acyl chains (C18:0, C20:0, C22:0, and C24:0) were increased in obese persons compared to the control group; they positively correlated with indicators of the blood serum lipid spectrum (TC, triacylglycerides, LDL-C) and the HOMA-IR insulin resistance index. Here, SM containing unsaturated acyl chains were not associated with these indicators.

However, Sigruener et al. [11] in their study of the plasma of over 3300 participants of the LUdwigshafen RISk and Cardiovascular Health (LURIC) study found that long-chain saturated SM with base d18:1 (C22:0; C23:0, C24:0) showed a negative relationship with CAD and overall death rate, i.e. they had cardioprotective properties. A moderate relationship with the protective effect was also found for SM with the C22:1 and C23:1 chains. On the contrary, SM with C16:0, C16:1, C24:1, and C24:2 chains showed a positive relationship with CAD and death rate.

Noteworthy results were obtained by Poss et al. [10] during the study of the relationship between sphingolipids in blood serum and CAD. Examining serum samples from people with the family CAD (n=462) and the control group (n=212), the authors demonstrated that SM(d18:1/16:0), SM(d18:1/18:0), SM(d18:1/20:0), SM(d18:1/22:0), SM(d18:1/24:0), SM(d18:1/24:1) were associated with CAD. Moreover, sphingolipids containing C24:1 (OR — 2.66; 95% CI: 2.12–3.38) were associated with CAD to the greatest extend.

Analysis of the literature data regarding the SM spectrum in patients’ AT indicates the ambiguity of earlier results. For instance, Barchuk et al. [2] conducted non-targeted lipid profiling of SAT and EAT in 25 patients with CAD and 14 individuals without CAD. The authors found a significant increase in the content of SM(d41:1) in the EAT of all participants (n=39), in contrast to the SAT. However, no nosology-depended differences in SM levels were found in EAT or SAT.

Tomášová et al. [12] conducted an MS-based lipidomic analysis of subcutaneous and epicardial AT in 23 patients with CAD and 13 control persons to identify factors in epicardial fat that contribute to the CAD development. The authors also did not find statistically significant differences in the content of SM(36:1), SM(40:1), SM(42:2) depending on the location of AT and coronary artery disease.

Kolak et al. [13] conducted a lipidomic analysis of SAT of 20 healthy obese nondiabetic women who were divided into groups with normal (n=10) and high (n=10) liver fat content. The following molecular SM types were increased in persons with high fat content: SM(d18:1/18:0), SM(d18:1/20:0), SM(d18:1/22:0), SM(d18:1/24:1). At that, the levels of the most common SM(d18:1/16:0) did not differ between groups. Given the increased expression of hypoxia-induced factor 1α (HIF-1α) and the trend towards increased expression of tumor necrosis factor α (TNF-α) in SAT of persons with high liver fat content, the authors concluded that long-chain fatty acids stimulate the synthesis of ceramides, which may cause AT inflammation.

The results of this study suggest an independent sphingomyelin profile of local fat depots (the same SM spectrum was identified in CAD and in AVHD) with a specific EAT enhancement with SM in case of coronarogenic pathology and in PVAT enhancement with SM in case of non-coronarogenic pathology.

Earlier, in [14] the authors showed a SM high concentration in plasma in case of atherosclerotic affected areas of the aorta. Examination of carotid plaques sampled within endarterectomy also revealed increased SM levels of in plaques associated with transient ischemic attacks, stroke, and stenosis >70% according to ultrasound. Moreover, SM levels correlated with inflammatory cytokines, histological markers of plaque instability, and plaque weight.

More recent MS-scanning studies found that SM amount to approximately 80% of phospholipids in necrotic affected areas of human atherosclerotic plaques [15]. The origin of atherosclerotic SM is still unclear. In the majority of studies, the observed changes are explained by increased uptake of SM-enhanced lipoproteins and their aggregation. At that, plaque LDL-C contains much more SM than plasma LDL-C [16].

According to the data available, PVAT of patients with AVHD is characterized by the highest level of all detected SM. The observed changes may result from inflammation, as aortic valve stenosis is also an inflammatory disease [17]. In a study of the aortic valves of 23 patients who underwent valve replacement surgery, Lehti et al. [17] demonstrated accumulation of extracellular lipid particles with a higher SM — phosphatidylcholine — in stenotic aortic valves compared with non-stenotic aortic valves. Studying the ascending thoracic aorta samples, Doppler et al. [18] revealed that, compared to controls, SM levels were significantly higher in patients with bicuspid aortic valve disease, who underwent ascending thoracic aorta surgery, and in persons with aortic dissection related to tricuspid aortic valve. The authors identified the following metabolites with significant differences between the control group and the groups with aneurysm of the ascending thoracic aorta: SM(C18:1), SM(C22:1), SM(C22:2), and SM(C24:1).

Thus, the comparative analysis conducted within this study provides that the sphingomyelin spectrum of local fat depots and blood serum is independent of nosology: similar SM spectrum was detected in CAD and in AVHD. However, SM content varied depending on the disease. In coronarogenic pathology, a specific AT enhancement with SM of epicardial localization was seen, in non-coronarogenic pathology — of perivascular localization. The observed changes may contribute to SM accumulation, triggering pathological processes associated with both CAD and AVHD. Moreover, the identified sphingomyelin profile of blood serum is wider than the SM spectrum in AT of various localizations, which may be due to formation of SM in the liver or endothelium of blood vessels.

The resulted new fundamental knowledge can become the basis for development of strategies for pharmacological correction of pathological activation of AT in patients with cardiac diseases, as well as of approaches to primary and secondary prevention of CVD. The established interconnections of indicators of the AT sphingomyelin profile with clinical and instrumental indicators in CVD evidence the relationship between the SM metabolism in AT of cardiac localization and disorders of systolic and diastolic function of the left ventricle in patients with CVD with multivessel coronary affected areas in CAD, as well as allow to use SM as promising biomarkers of CVD. Here, further research is required to establish the nature of these relationships.

### Limitations of the study

Firstly, this is a single-center study; secondly, the sample size was small; thirdly, studying other classes of lipids in local fat depots in patients with CVD is needed, which is planned as the future activity.

## Conclusion

Assessment of the blood lipid spectrum does not always properly reflect local dysfunctional changes in adipose tissue and prevents identification of all patients at high risk of cardiovascular diseases. Monitoring of changes in SM levels will allow to assess and anticipate development and/or severity of cardiovascular diseases, as well as to make them new therapeutic targets.
